# Resolution and Characterization of Distinct *cpn*60-Based Subgroups of *Gardnerella vaginalis* in the Vaginal Microbiota

**DOI:** 10.1371/journal.pone.0043009

**Published:** 2012-08-10

**Authors:** Teenus Paramel Jayaprakash, John J. Schellenberg, Janet E. Hill

**Affiliations:** 1 Department of Veterinary Microbiology, Western College of Veterinary Medicine, University of Saskatchewan, Saskatoon, Saskatchewan, Canada; 2 Department of Microbiology, University of Manitoba, Winnipeg, Manitoba, Canada; J. Craig Venter Institute, United States of America

## Abstract

Bacterial vaginosis (BV), characterized by a shift of the vaginal microbiota from a *Lactobacillus*-dominated community to a dense biofilm containing a complex mixture of organisms, is an important risk factor in poor reproductive health outcomes. The Nugent score, based on Gram stain, is used to diagnose BV and *Gardnerella vaginalis* abundance in the sample is one factor determining Nugent score. A high Nugent score is indicative of BV but does not always correspond to the presence of clinical symptoms. *G. vaginalis* is recognized as a heterogeneous group of organisms, which can also be part of the normal, healthy vaginal microbiome. In addition, asymptomatic BV and non-*Gardnerella* types of BV are being recognized. In an attempt to resolve the heterogeneous group of *G. vaginalis*, a phylogenetic tree of *cpn*60 universal target sequences from *G. vaginalis* isolates was constructed that indicates the existence of four subgroups of *G. vaginalis*. This subdivision, supported by whole genome similarity calculation of representative strains using JSpecies, demonstrates that these subgroups may represent different species. The *cpn*60 subgroupings did not correspond with the Piot biotyping scheme, but did show consistency with ARDRA genotyping and sialidase gene presence. Isolates from all four subgroups produced biofilm *in vitro*. We also investigated the distribution of *G. vaginalis* subgroups in vaginal samples from Kenyan women with Nugent scores consistent with BV, Intermediate and Normal microbiota (n = 44). All subgroups of *G. vaginalis* were detected in these women, with a significant difference (z = −3.372, n = 39, p = 0.001) in frequency of *G. vaginalis* subgroup B between BV and Normal groups. Establishment of a quantifiable relationship between *G. vaginalis* subgroup distribution and clinical status could have significant diagnostic implications.

## Introduction

*Gardnerella vaginalis,* first isolated by Leopold in 1953 [1], has long been recognized in vaginal samples and has been identified by several names, including *Haemophilus vaginalis* by Gardner and Dukes in 1955 [2]. Further characterization based on metabolic requirements and Gram staining led to its reclassification as *Corynebacterium vaginale* [3]. The proposal to create the genus *Gardnerella* and allocation of *Corynebacterium vaginale* and *Haemophilus vaginalis* to this new taxon as *Gardnerella vaginalis* was put forward by Greenwood and Pickett [4], based on a taxonomic study that utilized DNA-DNA hybridization, biochemical analysis of the cell wall, and electron microscopy.

*G. vaginalis* is strongly associated with bacterial vaginosis (BV), and is one of the most frequently isolated bacteria from women with symptoms of BV [5]–[7]. Abundance of *G. vaginalis* in vaginal samples has also been associated with infertility and preterm labour [8]. *G. vaginalis* has also been isolated from urine and blood and is associated with bacteremia, osteomyelitis and cervical cancer [9]–[11]. However, recent studies of vaginal microbiota indicate that *G. vaginalis* can also be a part of the vaginal microbiota in clinically healthy women [6], [12], [13].

*G. vaginalis* is recognized as a diverse taxon, both phenotypically and genotypically [13]–[15]. Eight biotypes of *G. vaginalis* have been identified by Piot *et al*. based on the presence of β-galactosidase, lipase and hippurate hydrolysis activities [14], whereas Benito *et al*. identified seventeen biotypes based on these characteristics in addition to fermentation of xylose, arabinose and galactose [15]. Phenotypic diversity within *G. vaginalis* has also been described in terms of virulence factors, particularly production of sialidase [16] and formation of biofilms [17]. Genetic heterogeneity within *G. vaginalis* has been demonstrated using amplified ribosomal DNA restriction analysis (ARDRA) [18]. Santiago *et al*. [16], identified three ARDRA genotypes of *G. vaginalis*, of which only two genotypes (genotypes 1 and 3) produced sialidase. However, like biotyping schemes, ARDRA can only be performed on isolates.

Genotype diversity is apparent in whole genome studies and in metagenomic studies of the human vaginal microbiome based on 16S rRNA [19] or *cpn*60 [6], [12]. Hummelen *et al.* [19] reported the presence of four types of *G. vaginalis* sequences differing by a single nucleotide within the 16S rRNA V6 region. Four clusters of *G. vaginalis* sequences, ranging between 89 and 100% sequence identity to the type strain (ATCC 14018^T^), were observed in a *cpn*60-based study of clinically healthy women by Hill *et al.* [12] and followed up in a larger study by Schellenberg *et al*. [6].

Previous work by our research group has demonstrated that *cpn*60 universal target sequences can resolve phenotypically distinct strains or ecotypes within an intestinal microbial community, and that these sequences are also excellent predictors of whole genome sequence relationships [20], [21]. The gene encoding the universal 60 kDa chaperonin protein (*cpn*60) is an established target for detection and identification of microorganisms, as well as gene-based metagenomic studies of complex microbial communities, including the vaginal microbiome [6], [12], [22]–[31]. An approximately 555 bp region corresponding to nucleotides 274–828 of the *E. coli cpn*60 gene can be amplified with degenerate, universal PCR primers [32], [33]. This universal target (UT) region is phylogenetically informative, providing more discriminating power than 16S rRNA to differentiate organisms, even at the sub-species or strain level [20], [34]–[42]. A highly curated reference database of chaperonin sequences, cpnDB, supports *cpn*60 based applications [43].

Given the observed phenotypic diversity (especially virulence factors), genotypic diversity, and the presence of *G. vaginalis* in women regardless of clinical status, it is critical to improve our understanding of the clinical significance of these different strains. In order to accomplish this most effectively and to lay the foundation for the development of more informative diagnostic tools for women’s health, direct culture-independent analysis of vaginal samples, exploiting a genetic target that facilitates robust resolution is required.

The objective of the current study was to investigate if previously observed *cpn*60 based subgroups of *G. vaginalis* are consistent with other (phenotypic) classification systems and/or available whole genome sequences, and to investigate the distribution of *cpn*60 defined subgroups of *G. vaginalis* in women with and without BV. Our results demonstrate that the *cpn*60 universal target sequence differentiates distinct subgroups within *G. vaginalis* and that only one of these subgroups (Subgroup B: Piot biotype 5, sialidase positive and ARDRA genotype 1) was found to be significantly more abundant in women with BV (high Nugent score) than women with normal vaginal microbiota in a retrospective analysis of metagenomic profiles of Kenyan women.

## Materials and Methods

### Bacterial Isolates

*G. vaginalis* ATCC 14018 (type strain) and ATCC 49145 were obtained from the American Type Culture Collection (Manassas, VA). Eight additional strains were isolated from Kenyan (N170, N165, N160, N158, N153, N148, N144, N143, N137, N134, N101, and N72) or Canadian (W11) women as described previously [6]. *G. vaginalis* isolates were cultured using the following media: ATCC #1685 broth (with or without 1% (w/v) glucose), Brain Heart Infusion broth (BHI) with 1% (w/v) glucose, egg yolk agar [14] and Columbia agar with 5% sheep blood (BD, Mississauga, ON). The GasPak EZ Pouch System (BD, Mississauga, ON) was used to provide anaerobic conditions.

### DNA Extraction and PCR

DNA was extracted from isolates using a phenol-chloroform extraction method and was stored at −20°C. Primers used in the study are listed in Table 1. *cpn*60 UT PCR amplicons were produced for direct sequencing, using universal primers H729 and H730 as described previously [33]. Primers JH0315 and JH0316 were designed based on the 16S rRNA sequence from *G. vaginalis* ATCC 14018. Amplification with these primers was carried out by incubating the reactions at 94°C for 3 minutes, followed by 40 cycles of 94°C for 30 sec, 52°C for 1 min and 72°C for 90 sec, and completed with a final extension of 10 min at 72°C. Sialidase gene presence was assessed by amplifying the sialidase gene using primers GVSI forward and GVSI reverse [16]. Vaginolysin gene sequences were amplified using primers V1 and V2 as previously described [44].

**Table 1:** Primers used in the study.

Primer name	Sequence (5′–3′)	Reference
H729	CGC CAG GGT TTT CCC AGT CAC GAC GAI III GCI GGI GAY GGI ACI ACI AC	[33]
H730	AGC GGA TAA CAA TTT CAC ACA GGA YKI YKI TCI CCR AAI CCI GGI GCY TT	[33]
JH0315	ATT CTG GCT CAG GAT GAA	This study
JH0316	GCT ACC TTG TTA CGA CTT AG	This study
GVSI forward	GAC GAC GGC GAA TGG CAC GA	[16]
GVSI reverse	AGT CGC ACT CCG CGC AAG TC	[16]
V1	ATG CAG CGA AGC ATG CCA TGC	[44]
V2	TCA GTC GTT CTT TAC AGT TTC	[44]
GV10F	GGT TCG ATT CTG GCT CAG	[16]
ωMB	TAC CTT GTT ACG ACT TCG TCC CA	[16]

### Phenotyping of *G. vaginalis* Isolates

Representative *G. vaginalis* isolates with unique *cpn*60 sequences were phenotyped using the Piot typing scheme using assays for hippurate hydrolysis, β-galactosidase and lipase activity as described previously [14]. *Lactobacillus crispatus* was used as a negative control for the lipase assay. *G. vaginalis* ATCC 14018^T^ was used as a positive control for all biochemical assays.

### Biofilm Formation

Isolates of *G. vaginalis* were cultured from −80°C stocks for 72 hrs, and subcultured for 48 hrs on Columbia sheep blood agar plates anaerobically at 37°C. A loopful of culture for each isolate was used to inoculate 4 ml of either ATCC broth #1685 with 1% (w/v) glucose or Brain Heart Infusion with 1% (w/v) glucose (BHIG) and incubated anaerobically for 48 hrs at 37°C. Broth cultures were diluted 1∶100 in media and 200 µL of diluted culture was added to individual wells of a 96-well tissue culture plate and incubated anaerobically for 48 hrs at 37°C. Qualitative assessment of biofilm formation was done by washing off the planktonic cells and staining the wells with 1% crystal violet solution to visualize any biofilm.

### ARDRA Genotyping

*G. vaginalis* isolates were genotyped using amplified rDNA restriction analysis (ARDRA) [16], [18]. Full-length 16S rRNA gene sequences were amplified using primers GV10F and ωMB (Table 1). PCR products were purified (QIAquick PCR Purification Kit, Qiagen, Inc., Toronto, ON) and subjected to overnight restriction digestion using TaqI (Life Technologies, Inc., Burlington, ON). The digestion products were resolved on a 1.5% agarose gel at 140 volts for 2 hrs. *In silico* ARDRA was performed for some strains for which only published genome sequence information was available (not the isolates themselves) by extracting full length 16S rRNA gene sequences from published whole genome sequences and then restricting the sequence using the program remap within the EMBOSS software suite [45].

### Sequence Sources

Published genome sequences of *G. vaginalis* used in the study, either completed or in progress, were downloaded from NCBI’s Genome database: strains 101 (Accession AEJD00000000), 315-A (AFDI00000000), 41V (AEJE00000000), 409-05 (CP001849), 5-1 (ADAN00000000), AMD (ADAM00000000), ATCC 14018 (ADNB00000000), ATCC 14019 (CP002104) and HMP9231 (CP002725). The metagenomic *cpn*60 sequences and *cpn*60 UT sequences of *G. vaginalis* isolates used were from a previously published study of vaginal microbiota of commercial sex workers in Kenya [6].

### Phylogenetic Analysis

*cpn*60 UT sequences obtained from whole genome sequences of *G. vaginalis* reference strains or amplified from cultured clinical isolates [6] were used to construct a phylogenetic tree, using *Alloscardovia omnicolens* CCUG 34444 as a root. Sequences were aligned using ClustalW (gap opening penalty = 10, gap extension penalty = 0.10) [46], followed by utilization of the Phylip software package [47] to calculate a distance matrix using dnadist and construct a tree using neighbor. The final tree was obtained from the bootstrapped consensus of 100 trees and was visualized using Dendroscope [48].

### Statistical Analysis

All statistical analyses were done using SPSS Statistics, version 19.0. For the analysis of assignment of assembled reads of Nairobi metagenomic data set to *G. vaginalis* subgroups, one-way ANOVA was done, followed by Post-hoc analysis by Tukey’s test. Statistical analysis for the distribution of *G. vaginalis* subgroups in Nairobi women was done by Kruskal-Wallis H test, followed by Mann-Whitney U test.

## Results

### *cpn*60-based Resolution of *G. vaginalis* Subgroups

**Figure 1:**
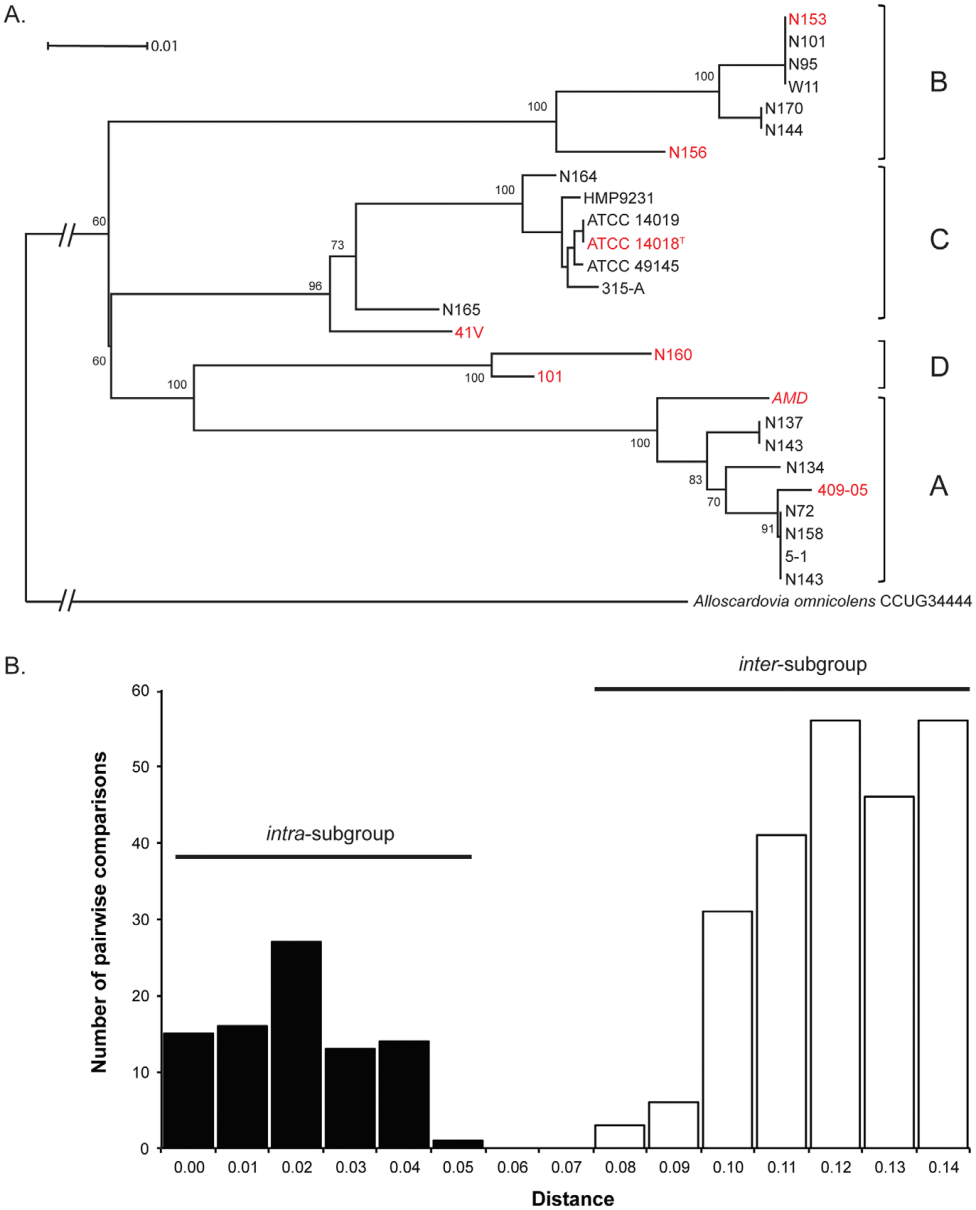
*cpn*60 UT sequence-based subgroups of *G. vaginalis*. A. Phylogenetic tree of *G. vaginalis*-like cpn60 UT sequences comprising four distinct clades: A, B, C and D. Bootstrap values for each node are indicated. 101, 315-A, 41V, 409-05, 5-1, AMD, ATCC 14018^T^, ATCC 14019, and HMP9231 are *G. vaginalis* isolates with whole genome sequence information available in Genbank (Accession numbers AEJD00000000, AFDI00000000, AEJE00000000, CP001849, ADAN00000000, ADAM00000000, ADNB00000000, CP002104 and CP002725 respectively). Isolates with names starting with “N” are isolates from Kenyan women from Schellenberg *et al.* [6]. W11 was isolated from a Canadian woman (Schellenberg, Unpublished). Sequences highlighted in red were used as representatives of the subgroups in the distribution analysis of metagenomic sequence data. B. Pairwise distances for the 26 *G. vaginalis* cpn60 UT sequences included in the phylogenetic analysis. Distances for both inter-subgroup comparisons (white bars) and intra-subgroup comparisons (black bars) are indicated.

A phylogenetic tree of *G. vaginalis cpn*60 UT sequences is shown in Figure 1A. This tree was used to select landmark sequences for further comparisons and for subgrouping of *G. vaginalis* metagenomic sequences. The discreteness of the subgroups was further supported by demonstration of a bimodal distribution of pairwise distances (inter- and intra-subgroup) between strains (Figure 1B). The four subgroups resolved were designated A, B, C and D and two representative sequences from each subgroup were selected as representatives to capture the maximum phylogenetic distance represented by the phylogenetic tree.

### Whole Genome, *cpn*60 and 16S rRNA Comparisons

Whole genome similarity calculations of representative strains of *G. vaginalis* for which there was either complete or partial genome sequence data available were calculated with JSpecies (Table 2). Within subgroups, pairwise Average Nucleotide Identity by MUMmer (ANIm) values were >95% and *cpn*60 identities were ≥96%, while between subgroups ANIm values were <90% and *cpn*60 identities were ≤92%. 16S rRNA pairwise identities were all 98–100%. Pairwise *cpn*60 and 16S rRNA sequence identities for representative isolates with unique *cpn*60 sequences are shown in Table 3. The pairwise sequence identity for *cpn*60 gene sequences and 16S rRNA gene sequences for isolates within *G. vaginalis* subgroups are >96% and >97%, respectively. Between *G. vaginalis* subgroups, pairwise identities ranged between 87% and 93% for *cpn*60 sequences and 97% and 100% for 16S rRNA sequences.

**Table 2:** ANIm (first row), cpn60 UT sequence identity (second row) and 16S rRNA sequence identity (third row) for representatives of *G. vaginalis* subgroups A, C and D, for which whole genome sequence data was available.

		A		C					D
		AMD	5-1	ATCC 14019	41V	ATCC 14018	315-A	HMP9321	101
	409-05	95.86	98.2	89.09	88.97	89.13	88.85	89.1	88.56
		97	99	88	90	88	88	88	91
		99	100	98	98	98	98	98	99
A	AMD		95.7	89.06	88.64	89	88.98	89.59	88.51
			97	89	90	89	88	89	91
			99	98	98	98	98	98	99
	5-1			89.31	88.89	89.29	88.96	89.96	88.91
				88	91	88	88	88	91
				98	98	98	98	98	99
	ATCC 14019				95.91	99.79	98.19	98.35	88.29
					96	100	99	99	92
					99	100	100	100	98
	41V					95.87	96.04	96.07	88.66
						96	96	96	92
						99	99	99	98
C	ATCC 14018						98.13	98.27	88.22
							99	99	92
							100	100	98
	315-A							98.38	88.45
								99	92
								100	98
	HMP9321								89.19
									92
									98

No whole genome sequence is available for a subgroup B strain.

**Table 3:** Pairwise sequence identity of cpn60 (first row of each column) and full-length 16S rRNA (second row) for *G. vaginalis* isolates.

	A					B			C								D	
	N158	N134	AMD	5-1	N137	N156	N153	N144	ATCC14019	N165	N164	41V	ATCC14018	315-A	HMP9231	ATCC49145	N160	101
409-05	99	98	97	99	98	90	87	87	88	90	89	90	88	88	88	88	90	91
	99	99	99	100	99	ND	99	99	98	98	ND	98	98	98	98	98	99	99
N158		98	97	100	98	90	87	87	88	90	89	91	88	88	88	88	90	91
		99	99	99	99	ND	99	99	98	98	ND	98	98	98	98	98	99	99
N134			97	98	98	89	87	88	88	90	89	90	88	88	88	88	90	91
			99	99	99	ND	99	99	98	98	ND	98	98	98	98	98	99	99
AMD				97	97	89	87	88	89	91	89	90	89	88	89	89	90	91
				99	100	ND	99	99	98	98	ND	98	98	98	98	98	99	99
5-1					98	90	87	87	88	90	89	91	88	88	88	88	90	91
					99	ND	99	99	98	98	ND	98	98	98	98	98	99	99
N137						89	87	88	89	91	90	91	89	89	89	89	90	91
						ND	99	99	98	98	ND	98	98	98	98	98	99	99
N156							96	96	88	90	89	89	88	89	89	88	88	88
							ND	ND	ND	ND	ND	ND	ND	ND	ND	ND	ND	ND
N153								98	89	90	89	89	89	90	89	89	88	88
								99	99	99	ND	99	99	99	99	99	99	99
N144									89	91	90	90	89	90	90	90	88	88
									99	99	ND	99	99	99	99	99	99	98
ATCC14019										97	99	96	100	99	99	99	90	92
										99	ND	99	100	100	100	99	98	98
N165											97	97	97	96	96	96	91	93
											ND	99	99	99	99	99	98	98
N164												96	99	98	99	98	91	92
												ND	ND	ND	ND	ND	ND	ND
41V													96	96	96	96	91	92
													99	99	99	99	98	98
ATCC14018														99	99	99	90	92
														100	100	99	98	98
315-A															99	99	90	92
															100	99	98	98
HMP9231																99	91	92
																99	98	98
ATCC49145																	90	92
																	98	98
N160																		98
																		99

ND = Not done. Only representative study isolates with unique *cpn*60 sequences are included.

### Biotyping, Sialidase, ARDRA Genotyping and Biofilm Production

**Figure 2:**
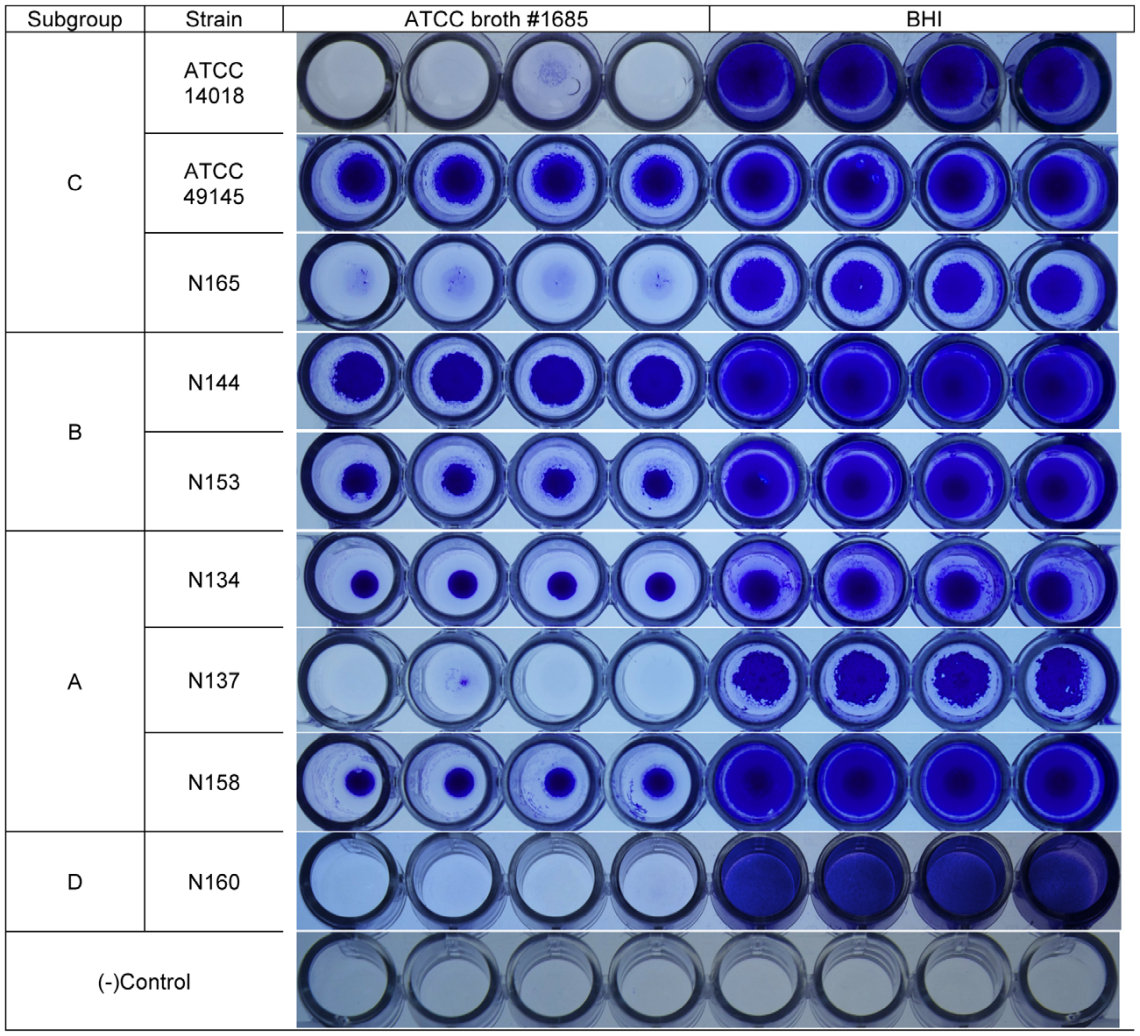
Biofilm formation by *G. vaginalis.* Isolates were cultured in 96-well plate in two different media: ATCC broth #1685 and BHI, stained at 48 hrs, after removal of planktonic cells.

Results for the biotyping assays and genotyping are shown in Table 4. Piot biotypes 1, 2, 5, 7 and 8 were identified among the isolate collection, but no consistent pattern of biotype distribution and *cpn*60 subgroup was observed. All subgroup C isolates were lipase positive. Subgroup B and C isolates were sialidase gene positive and ARDRA genotype 1, whereas subgroup A isolates were genotype 2 with no detection of the sialidase gene. Subgroup D isolates differed in sialidase gene presence (N160 was negative, strain 101 positive) and ARDRA genotype (N160 was genotype 2, strain 101 was predicted to be genotype 1 based on *in silico* restriction analysis). All isolates produced biofilm in BHIG by 48 hrs, and substantial variability in biofilm production was observed in the two media tested (BHIG and ATCC broth #1685 with 1% glucose) (Figure 2). Both subgroup B isolates (N144 and N153) formed biofilm in both media, although the biofilm formed in BHIG was more extensive, completely coating the well. In subgoups A and C, at least one of the isolates failed to produce any visible biofilm in ATCC broth #1685.

**Table 4:** Piot biotype, sialidase gene presence, and ARDRA characterization of *G. vaginalis* isolates (representatives of study isolates with unique *cpn*60 sequences) and published whole genome sequences.

*cpn*60 subgroup	Isolate^2^	Piot Biotype^1^				Sialidase gene	ARDRA^3^
		L	B	H	Biotype		
C	41V	ND	ND	ND	ND	+	1
	N165	+	+	−	8	+	1
	ATCC 14018	+	+	+	1	+	1
	ATCC 49145	+	+	−	8	+	1
	ATCC 14019	ND	ND	ND	ND	+	1
B	N144	−	−	+	5	+	1
	N153	−	−	+	5	+	1
D	101	ND	ND	ND	ND	+	1
	N160	−	−	+	5	−	2
A	AMD	ND	ND	ND	ND	−	2
	N137	−	−	−	7	−	2
	N134	−	−	+	5	−	2
	409-05	ND	ND	ND	ND	−	2
	5-1	ND	ND	ND	ND	−	2
	N158	+	−	+	2	−	2

1 L = Lipase, B = β-galactosidase, H = Hippurate hydrolase, ND = not done.

2 Study isolates N156 (subgroup B) and N164 (subgroup C) were not included in the biotyping analysis since they were not reliably cultured as pure isolates after revival from frozen stocks following their original isolation and *cpn*60-based characterization.

3 In cases where no isolates were available to us for culture, ARDRA genotypes for some strains (41V, ATCC 14019, 101, AMD, 409-05, and 5-1) were obtained by *in silico* analysis as described in the text.

We also attempted to detect vaginolysin gene sequences in the isolates selected for phenotypic analysis. A PCR product of the expected size of 1,551 bp was obtained for only three isolates (ATCC 14018^T^, ATCC 49145 and N153). An amplicon of 1,200 bp was amplified from three others (N134, N137 and N158), but sequence analysis indicated a mixture of products, suggesting that this product was the result of non-target sequence amplification. Isolates N165 and N144 did yield any product after repeated attempts. The vaginolysin sequence from ATCC 49145 was 99% identical to ATCC 14018 and only 91% identical to N153.

### Distribution of *G. vaginalis* Subgroups in Kenyan Women

**Figure 3:**
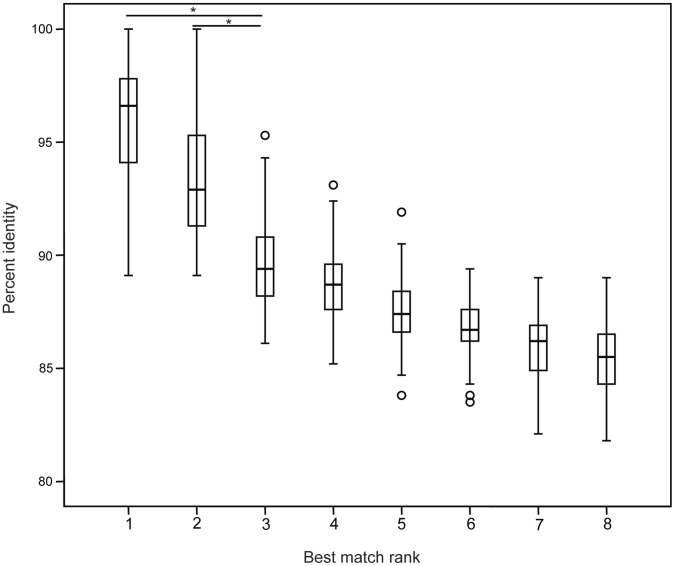
Percent identity of metagenomic sequences to *G. vaginalis* reference strains. Distribution of percent identity of *G. vaginalis* metagenomic sequences to their first through eighth best matches among representative sequences of the *G. vaginalis* subgroups. The reference database included two representatives of each subgroup, indicated in Figure 1. Significant differences in percent identity (*p*<0.0001) are indicated by *.

A previously published *cpn*60 metagenomic dataset was used to investigate distribution of *cpn*60-based *G. vaginalis* subgroups in vaginal microbiome profiles derived from samples classified as BV, Intermediate or Normal based on Nugent score [6]. All unique sequences assembled from the study data (n = 831 OTU) were compared using watered-BLAST [30] to a reference database of *cpn*60 sequences containing one representative of each species in cpnDB (cpnDB_nr; www.cpndb.ca) and two representatives of each *G. vaginalis* subgroup as indicated in Figure 1. All assembled sequences with any of the *G. vaginalis* reference sequences as their best match, and meeting the minimum requirement for identification as a *cpn*60 sequence (60% identity over ≥100 nucleotides) were included in the analysis of distribution (n = 93). For 84/93 of the assembled metagenomic sequences, the top two hits were to the same *G. vaginalis* subgroup. Identities for each query and its top two hits (medians for 93 queries were 96.6% and 92.9% respectively) were significantly (*p*<0.0001) higher than identity to the third (median 89.4%) through eighth best hits (Figure 3). Thirty-one queries had sequence identities <95% to their best match.

**Figure 4:**
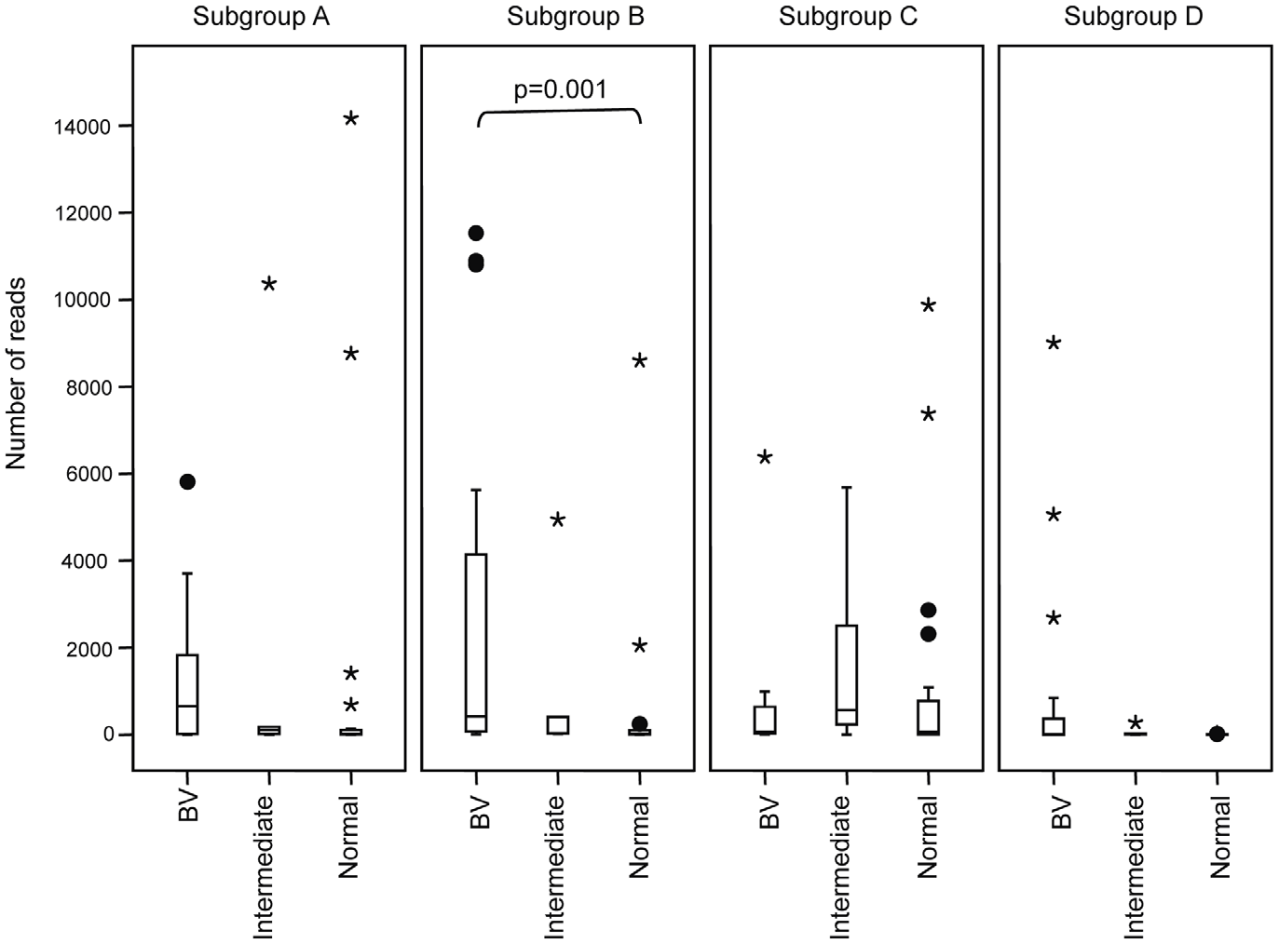
Distribution of *G. vaginalis* subgroups in African women. Relative abundance of sequence reads corresponding to *Gardnerella* subgroups (scaled to median library size of 15,000 reads) among clinical categories (BV, Intermediate and Normal, based on Nugent score). Boxplots were created for each *Gardnerella* subtype and p values calculated based on non-parametric significance tests (Mann-Whitney U test) using SPSS Statistics version 19.0.

Once metagenomic sequences were assigned to a subgroup (based on the best watered-BLAST match) the distribution of the subgroups among the vaginal microbiomes of women diagnosed as BV (n = 20), Intermediate (n = 5) and Normal (n = 19), based on Nugent score was determined (Figure 4). The sequence read frequencies used in this analysis were normalized to the median library size of 15,000 reads [6]. All vaginal microbiota libraries contained sequences corresponding to more than one subgroup of *G. vaginalis*. Out of 44 libraries sequenced, 41, 43, 43 and 27 contained *G. vaginalis* subgroup A, B, C and D respectively. The majority of libraries (25/44) contained sequence from all four subgroups. The next most prevalent combination was A+B+C (n = 14 libraries), while other combinations were present in the remaining four samples (n = 1 for A+C, n = 2 for B+C, n = 1 for B+C+D, n = 1 for A+B+D). The difference in frequencies of *G. vaginalis* subgroup sequence reads was tested using Kruskal-Wallis H test (SPSS Statistics, version 17.0). A significant difference (χ^2^ value = 12.329, df = 2, *p* = 0.002) was observed only for subgroup B sequences. Pairwise comparison on subgroup B sequences between the clinical groups (BV, Intermediate, and Normal) was analyzed by Mann-Whitney U test and results showed a significantly greater abundance of subgroup B sequences in BV compared to Normal (z = −3.372, n = 39, *p* = 0.001).

## Discussion

Bacterial vaginosis is the most commonly reported vaginal infection [49], [50]. BV can be diagnosed clinically using Amsel’s criteria, which include presence of homogenous vaginal discharge, a vaginal pH of greater than 4.5, positive whiff test (production of a fishy odour on addition of 10% KOH to vaginal sample), and also presence of clue cells in at least 20% of the total cell count [51]. The Nugent score is another commonly used diagnostic tool for BV. To calculate the Nugent score, a Gram stained vaginal smear is assessed for the relative abundance of various bacterial morphotypes including Gram-positive large rods, Gram-negative/Gram-variable rods and curved Gram-variable rods. With increasing numbers of survey studies in which Nugent scores of clinically normal women are determined, the phenomenon of “asymptomatic BV” has been widely observed [52]–[54]. These women have high Nugent scores, but do not have symptoms of BV. The clinical significance of asymptomatic BV is unknown. Since the presence of *G. vaginalis* is one of the key determinants of Nugent score, one possible explanation for asymptomatic BV is the presence of large numbers of non-pathogenic *G. vaginalis* or other species with similar Gram stain morphology. If this is true, then the detection of *G. vaginalis* in general may be of questionable diagnostic value. Resolution of this important issue requires the investigation of distribution of *G. vaginalis* lineages among women in a variety of clinical cohorts. Tackling this on a large scale requires culture-independent tools that provide resolution of phenotypically distinct *G. vaginalis* subgroups when applied directly to clinical samples.

Sequence diversity within *G. vaginalis* has been reported in metagenomic studies of the vaginal microbiome based on 16S rRNA and cpn60 gene targets, and four subdivisions have been reported in several recent studies [6], [12], [19]. The subdivision observed by Hummelen et al. [19] was based on single nucleotide differences within the V6 region of 16S rRNA. The much lower *cpn*60 sequence identity between these subgroups (≤93% versus ≥98% identity for 16S rRNA) facilitated the identification of vaginal isolates corresponding to these subgroups, demonstrating that the metagenomic studies had revealed real biological diversity and not artifactual diversity resulting from PCR, sequencing and or data assembly (Figure 1). Overestimation of microbial diversity in metagenomic sequencing studies is an ongoing concern [55], so the identification and characterization of actual isolates corresponding to metagenomic sequences is reassuring and further supports the value of the *cpn*60 universal target for resolution of diversity at species- and strain-level [20].

Whole genome DNA–DNA hybridization persists as the gold standard method for defining bacterial species [56] although it remains unpopular due to its technical demands [57]. DNA sequence data is increasingly relied upon to support species definition and resolution, and recently whole genome sequence comparison has been suggested as a new gold standard [58]. Richter & Rosselló-Mora [58] demonstrated that average nucleotide identity (ANI) values correlate well with DNA-DNA hybridization results and suggest that an ANI values greater than ≈95–96%, calculated by either BLAST or the MUMmer rapid aligning tool, were indicative of bacteria of the same species. Another alternative was proposed by Ziegler, who developed a computational algorithm based on sequence of three genes (recN, rpoA and thdF) that corresponds well to the conclusions of DNA-DNA hybridization data [59]. In that study, the 16S rRNA gene, widely used for identifying bacterial species and metagenomic studies, was found to have the lowest correlation between sequence identity and genome sequence identity. Subsequently, Verbeke *et al.* [21] demonstrated that a *cpn*60 UT sequence alone could predict whole genome identity as well as the three gene model. The ease of amplifying and sequencing the *cpn*60 UT from bacteria, the curated reference database of chaperonin sequences (cpnDB, www.cpndb.ca) [43], and the ability of the *cpn*60 UT to predict whole genome sequence similarity make it the ideal target for studies of *Gardnerella*, or any other bacterial taxon for which subspecies resolution is of interest.

Our results show a strong relationship between *cpn*60 UT sequence identity and whole genome comparison with the ANIm algorithm in JSpecies (Table 2). In fact, our results suggest that subgroups A, C and D of *G. vaginalis* meet the whole genome sequence-based criteria for designation as different species. Although no whole genome sequence is available for a subgroup B isolate, the *cpn*60 sequence data for isolates in this group certainly support a similar species level status for this group (Table 3). Complete genomes of nine *G. vaginalis* strains determined at the time of writing, and the fact that none of them belongs to subgroup B is interesting. Our experience with culturing of subgroup B isolates suggest that their conspicuous absence from the genome sequence database is most likely due to the fact that members of this subgroup, unlike the others, only grow in anaerobic conditions and do not grow in 7% CO_2_, which is the atmosphere recommended for routine isolation of *G. vaginalis* [60].

The heterogeneity of the *G. vaginalis* taxon is well documented based on application of biotyping schemes. Some of the biotypes of *G. vaginalis* from both the Piot and Benito biotyping schemes have been associated with BV [61], [62]. Piot biotypes 1, 4 and 5 are the most frequently isolated regardless of BV status [13] and biotype 5 has been reported to be predominantly associated with healthy vaginal ecosystems [61]. Piot biotypes 7 and 8 have been reported as the most frequently isolated from BV patients with isolation rate of 32% and 20%, respectively [61]. Of the seven isolates characterized in this study, four were Piot biotype 5, supporting previous observations of the prevalence of this biotype (Table 4). Otherwise, biotyping results were not consistent with sialidase gene presence, ARDRA or observations of association with BV in the Kenyan cohort. Although we did not provide evidence of sialidase enzymatic activity in our isolates, we observed a consistent relationship between the presence or absence of the sialidase gene and ARDRA genotype in that we detected the sialidase gene in all genotype 1 isolates examined and did not detect the sialidase gene in any of the genotype 2 isolates examined. Sialidase activity is recognized as a virulence factor in *G. vaginalis* and is the basis for a chromogenic, BV Blue Kit for BV diagnosis [63], but the results of reports examining the correlation between sialidase gene presence and detection of sialidase enzymatic activity are variable, making it inadvisable to draw conclusions about sialidase activity based solely on gene presence [16], [64].

Vaginolysin is a protein toxin belonging to the cholesterol-dependent cytolysin family of toxins that has been previously identified in *G. vaginalis* [44]. To detect this purported virulence factor in the study isolates, we employed previously published PCR primers designed based on the type strain, ATCC 14018. The primers failed to amplify the target sequence from most study isolates, and among the isolates for which we did generate sequence (ATCC 14018, ATCC 49145 and N153), we observed only 91% nucleotide sequence identity between some isolates (N153 vs. either the type strain or ATCC 49145). These results suggest that these primers may be too specific for general application in *G. vaginalis,* rather than indicating the absence of a vaginolysin gene in the other strains included in the study.

Subgroups of *G. vaginalis* were not evenly distributed among vaginal microbiomes diagnosed as BV, intermediate or normal based on Nugent score (Figure 4). Although *G. vaginalis* sequences were ubiquitous in the study group, and most women hosted multiple subgroups of *G. vaginalis*, only subgroup B was significantly more abundant in BV than normal samples. Analysis of pH and clue cells in these samples showed, as expected, a negative correlation of pH and Nugent score and a positive correlation of clue cells and Nugent score (data not show). An obvious and immediate question is whether subgroup B or any other subgroup is differentially associated with symptomatic and asymptomatic BV. Although we were unable to stratify our current data by symptoms (discharge, odour), relatively low *cpn*60 sequence identities facilitate robust differentiation of *G. vaginalis* subgroups (Figure 1 and 3) making it an ideal target to exploit in culture-independent approaches to addressing these questions in future studies. High throughput sequencing of *cpn*60 amplicons [30], bead-based hybridization assays [65] and quantitative real-time PCR methods [66] have all been developed based on *cpn*60 UT sequences and offer powerful tools for investigation of microbial diversity at, and below, the species level.

The results of our work support previous observations of genotypic and phenotypic diversity within *G. vaginalis* and we have been successful in using *cpn*60 UT sequences for robust classification of available *G. vaginalis* strains into four subgroups. We have also provided evidence that supports the eventual reclassification of subgroups as different species of *Gardnerella*. The degree of *cpn*60 UT and whole genome sequence diversity within this taxon is beyond that associated with “ecotypes” [67] or strains and suggests that reclassification may be warranted. However, additional genotypic and phenotypic analysis of additional isolates will be required to make this case. The cpn60 UT sequence offers a robust tool for identification of subgroups within *G. vaginalis* that may not be discernable using other targets. This feature of the *cpn*60 target will facilitate future efforts to expand diagnostic panels for rapid, high throughput characterization and improved resolution of species and strain distribution in the vaginal microbiome.
